# 

**Published:** 1872-06

**Authors:** 


### ATLANTA MEDICAL AND SURGICAL JOURNAL.

EDITED BY

JOSEPH P. LOGAN, M.D.,

AND

W. F. WESTMORELAND, M.D.,

*Professor of the Principles and Practice of Surgery in Atlanta Medical College.*

VOL. X.—JUNE, 1872.—NO. 3.

*PAX ET SCIENTIA, SED VERITAS SINE TIMORE.*

ATLANTA, GEORGIA:
PLANTATION PUBLISHING COMPANY'S PRESS.
1872.

